# Performance Characterization of the Smartphone Video Guidance Sensor as Vision-Based Positioning System

**DOI:** 10.3390/s20185299

**Published:** 2020-09-16

**Authors:** Nasir Hariri, Hector Gutierrez, John Rakoczy, Richard Howard, Ivan Bertaska

**Affiliations:** 1 Department of Mechanical and Energy Engineering, College of Engineering, Imam Abdulrahman Bin Faisal University, P.O. Box 1982, Dammam 31441, Saudi Arabia; nghariri@iau.edu.sa; 2 Mechanical and Aerospace Engineering, Florida Institute of Technology, Melbourne, FL 32901, USA; 3 Control Systems Design and Analysis Branch, NASA Marshall Space Flight Center, Huntsville, AL 35812, USA; john.m.rakoczy@nasa.gov (J.R.); ivan.r.bertaska@nasa.gov (I.B.); 4 Avionics Subsystems Branch, NASA Marshall Space Flight Center, Huntsville, AL 35812, USA; ricky.howard@nasa.gov

**Keywords:** video sensor, guidance, navigation, motion control, photogrammetry, attitude, spacecraft proximity maneuvers, rendezvous, docking

## Abstract

The Smartphone Video Guidance Sensor (SVGS) is a vision-based sensor that computes the six-state position and orientation vector of a target relative to a coordinate system attached to a smartphone. This paper presents accuracy-characterization measurements of the Smartphone Video Guidance Sensor (SVGS) to assess its performance as a position and attitude estimator, evaluating its accuracy in linear and angular motion for different velocities and various types of targets based on the mean and standard deviation errors between SVGS estimates and known motion profiles, in both linear and angular motions. The study also examines the effects of target velocity and sampling rate on the overall performance of SVGS and provides an overall assessment of SVGS’ performance as a position/attitude estimator. While the error metrics are dependent on range and camera resolution, the results of this paper can be scaled to other operational conditions by scaling the blob size in pixels (the light markers identified in the images) relative to the total resolution (number of pixels) of the image. The error statistics of SVGS enable its incorporation (by synthesis of a Kalman estimator) in advanced motion-control systems for navigation and guidance.

## 1. Introduction

Vision-based positioning systems [1,2,3,4,5,6] have been explored as an alternative for the autonomous navigation of spacecraft and other robotics applications. The Smartphone Video Guidance Sensor (SVGS) is a photogrammetric embedded sensor developed at NASA Marshall Space Flight Center using an Android-based smartphone [7,8] for support of proximity operations and formation flight maneuvers in small satellites. SVGS estimates the relative position and orientation of a moving target relative to a coordinate system attached to the camera by capturing an image of a set of retroreflective or illuminated targets mounted on the target in a known geometric pattern. The image is processed using a modification of algorithms originally developed for the Advanced Video Guidance Sensor (AVGS) [9,10,11], which successfully flew on the Demonstration for Autonomous Rendezvous Technology (DART) and Orbital Express missions [12,13]. SVGS can be deployed in a variety of robotic platforms by using a camera and CPU available in the target, and is part of the development at NASA Marshall Space Flight Center of a low-cost, low mass, system that enables navigation within proximity distance between small satellites, enabling formation flight and autonomous rendezvous and capture (AR & C) maneuvers. AVGS is capable of estimating the full six-degrees-of-freedom relative position and attitude vector in the near range, and further developments are being investigated to expand its capabilities for long-range proximity operations [9,14]. SVGS is similar to AVGS in that both use photogrammetric techniques to calculate the 6 × 1 position and attitude vector of the target. However, the hardware and deployment scenarios of AVGS are significantly different from those of SVGS [7,8,9,10,11]. SVGS has not been deployed in space missions yet, but NASA foresees a significant role for SVGS as a leading technology in future rendezvous, docking and proximity operations—a demonstration mission for SVGS on board the International Space Station is currently ongoing. Furthermore, SVGS has potential to be used in a variety of robotic applications where proximity operations, landing, the coordination of agents and docking is needed and can therefore be of potential use to a larger community. “Near range” in SVGS can be somewhere between a few meters to up to 200 m depending on the application: The target dimensions need to be adjusted to fit the required range for a given application. This paper presents a performance assessment of SVGS as a position and attitude sensor for proximity operations. The error statistics of SVGS enable its incorporation (by synthesis of a Kalman estimator) in advanced motion control systems for navigation and guidance.

**Figure 1:**
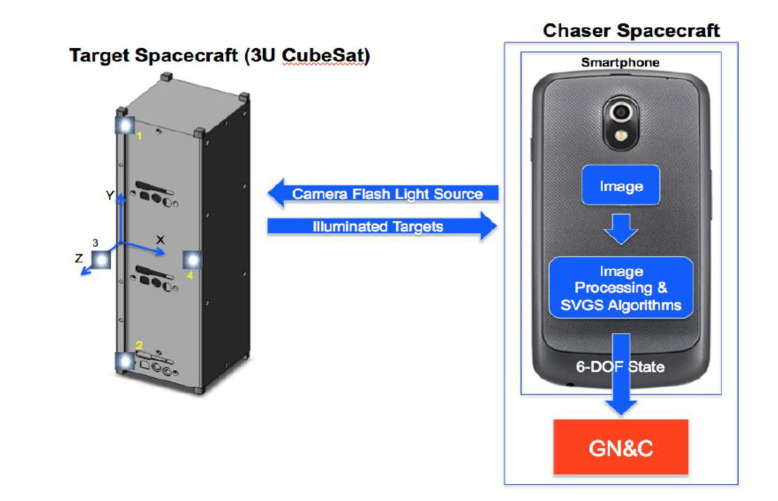
The operational concept of Smartphone Video Guidance Sensor (SVGS) [7]. The target’s six-degrees-of-freedom (DOF) state can be transmitted from the SVGS device to the spacecraft’s guidance, navigation and control system (GN & C).

Figure 1 shows the operational concept of SVGS. Estimating the target’s position and attitude relative to the camera’s coordinate system starts with the image capture of a set of illuminated targets. The six-degrees-of-freedom position and attitude vector are estimated using geometric photogrammetry techniques [7,8,15], where all image processing and state estimation are performed onboard the smartphone, alleviating the computational load on a motion control computer.

The basic rendition of the SVGS sensor uses a smartphone camera (Figure 1) to identify a known pattern of retro-reflective or illuminated targets placed on a target spacecraft. An image of the illuminated targets is captured, and using simple geometric techniques, the six-degrees-of-freedom (DOF) position and attitude state are extracted from the two-dimensional image. While AVGS used a laser as its illumination source and a high-quality CMOS sensor to capture the images, SVGS [7,8] uses the camera and flash on a generic Android smartphone. SVGS is a low-mass, low-volume, low-cost implementation of AVGS, designed for application on CubeSats and other small satellites to enable autonomous rendezvous and capture (AR & C) and formation flying.

In SVGS, the complete state calculation, including image capture, image processing and relative state derivation is performed on the Android device, and the 6-DOF relative state is calculated. The computed state can then be used by other applications or passed from the phone to other avionics onboard a small satellite as input data for the guidance, navigation and control functions.

**Target Pattern and Coordinate System**. The target pattern used for SVGS is a modified version of the AVGS pattern. A target example is shown on a 3U CubeSat mockup in Figure 1. Three illuminated targets are mounted coplanar (at the edges of the long face of the CubeSat), while a fourth is mounted on a boom. By placing the fourth illuminated target out of plane relative to the others, the accuracy of the relative attitude calculations is increased. The SVGS target spacecraft coordinate system is defined as follows: (i) the origin of the coordinate system is at the base of the boom, (ii) the *y*-axis points along the direction from target 2 to target 1, (iii) the *z*-axis points from the origin towards target 3, and (iv) the *x*-axis completes the right-handed triad. The 6-DOF position/attitude vector calculated by the SVGS algorithm is defined in a coordinate system with the same orientation as above but with the origin located at the center of the image plane formed by the smartphone camera.

**Figure 2:**
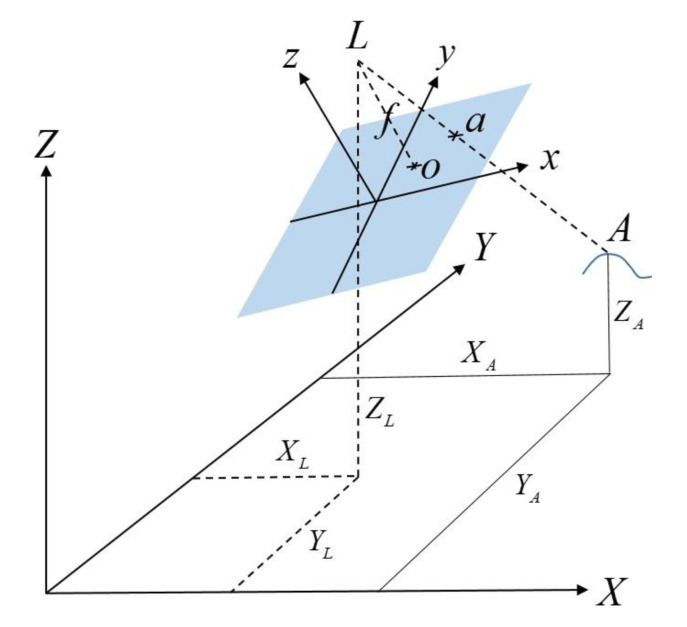
Object and camera frame geometry of SVGS. The image plane is shown in blue.

AVGS uses the Inverse Perspective algorithm [10] to calculate the 6-DOF relative state between the target and chase vehicles. SVGS, on the other hand, uses photogrammetry techniques [13,14] and an adaption of the collinearity equations developed by Rakoczy [8] to solve for the desired state vector. If a thin lens camera system captures point A as shown in Figure 2 [8], all light rays leaving point A and entering the camera are focused at point L (called the perspective center), at the location of the camera lens. An image of point A on the image plane is represented as point “a”; the image plane is located a distance *f* from the perspective center, where *f* is the focal length of the lens. Figure 2 shows two coordinate frames: the object (or target) frame <*X*, *Y*, *Z*>, and the image (or chase) frame <*x*, *y*, *z*>. A vector from the perspective center to point “A” can be defined in the object frame as *v_A_*, while a vector to point “a” from the perspective center is *v_a_*:

(1) $$v_{A}\;=\;\left[\begin{array}{c} X_{A}\;-\;X_{L}\\ Y_{A}\;-\;Y_{L}\\ Z_{A}\;-\;Z_{L} \end{array}\right]\begin{array}{ccc} & ; & \end{array}v_{a}\;=\;\left[\begin{array}{c} x_{a}\;-\;x_{0}\\ y_{a}\;-\;y_{0}\\ -f \end{array}\right]$$

These two vectors are related by Equation (2), where k is a scaling factor and M is a rotation matrix representing an x, y, z rotation sequence transforming the object frame to the image frame:

(2) $$v_{a}\;=\;kMv_{A}$$

Dropping the “*a*” and “*A*” subscripts and solving for the image frame coordinates *x*, *y* and *z* of point “a”, followed by dividing by “*z*” to eliminate the scaling factor *k*, yields the following two equations for *F_x_* and *F_y_*, where the *m_ij_* values are elements of the direction cosine matrix *M*:

(3) $$x\;=\;f\frac{m_{11}\left(X\;-\;X_{L}\right)\;+\;m_{12}\left(Y\;-\;Y_{L}\right)\;+\;m_{13}\left(Z\;-\;Z_{L}\right)}{m_{31}\left(X\;-\;X_{L}\right)\;+\;m_{32}\left(Y\;-\;Y_{L}\right)\;+\;m_{33}\left(Z\;-\;Z_{L}\right)}\;+\;x_{0}\;=\;F_{x}$$

(4) $$y\;=\;f\frac{m_{21}\left(X\;-\;X_{L}\right)\;+\;m_{22}\left(Y\;-Y_{L}\right)\;+\;m_{23}\left(Z\;-\;Z_{L}\right)}{m_{31}\left(X\;-\;X_{L}\right)\;+\;m_{32}\left(Y\;-\;Y_{L}\right)\;+\;m_{33}\left(Z\;-\;Z_{L}\right)}\;+\;y_{0}\;=\;F_{y}$$

The relative 6-DOF state vector that needs to be solved for is *V* is defined below, where *ϕ*, *θ* and *ψ* represent the *x*, *y* and *z* rotation angles, respectively:

(5) $$V\;=\;{\left[\begin{array}{cccccc} X_{L} & Y_{L} & Z_{L} & \phi & \theta & \psi \end{array}\right]}^{T}$$

Linearizing *F_x_* and *F_y_* using a Taylor series expansion truncated after the second term yields:

(6) $$x\;=\;F_{x}(V_{0})\;+\;\frac{\partial F_{x}}{\partial V}\Delta V\;+\;\varepsilon_{x}$$

(7) $$y\;=\;F_{y}(V_{0})\;+\;\frac{\partial F_{y}}{\partial V}\Delta V\;+\;\varepsilon_{y}$$

where *V*_0_ is an initial guess for the state vector, and Δ*V* is the difference between this guess and the actual state vector:

(8) $$\Delta V\;=\;V\;-\;V_{0}$$

*ε_x_* and *ε_y_* are the *x* and *y* error due to the Taylor series approximation. Each of the four targets in the SVGS target pattern has a corresponding set of these two equations; the resulting eight equations can be represented in matrix form using the following notation:

(9) $$Y\;=\;\left[\begin{array}{c} x_{1}\\ y_{1}\\ ..\\ ..\\ x_{4}\\ y_{4} \end{array}\right]\begin{array}{cc} ; & \end{array}Y_{0}\;=\;\left[\begin{array}{c} F_{x_{1}}\\ F_{y_{1}}\\ ..\\ ..\\ F_{x_{4}}\\ F_{y_{4}} \end{array}\right]\begin{array}{cc} ; & \end{array}H\;=\;{\left[\begin{array}{cccccc} \frac{\partial F_{x_{1}}}{\partial V} & \frac{\partial F_{y_{1}}}{\partial V} & .. & .. & \frac{\partial F_{x_{4}}}{\partial V} & \frac{\partial F_{y_{4}}}{\partial V} \end{array}\right]}^{T}$$

(10) $$Y\;=\;Y_{0}\;+\;HV\;+\;\varepsilon$$

This equation is solved for the V that minimizes the square of the residuals ε. This value is then added to the initial estimate of *V* to get the updated state vector. The process is iterated until the residuals are sufficiently small, yielding the final estimate of the 6-DOF state vector *V*.

**SVGS Collinearity Formulation**. In SVGS, the general form of the collinearity equations described above is narrowed down to reflect the state vector formulation used by AVGS. AVGS sensor measurements used angle pairs, azimuth and elevation, measured in the image frame to define the location of each retro-reflective target in the image. Azimuth and elevation are measured with respect to a vector to the perspective center and the target locations in the captured image. Azimuth, *A_z_*, (Equation (11)), and elevation, *E_l_* (Equation (12)), replace Equations (3) and (4) to yield:

(11) $$A_{z}\;=\;\tan^{-1}\left(\frac{x\;-\;x_{0}}{f}\right)$$

(12) $$E_{l}\;=\;\sin^{-1}\left(\frac{y\;-\;y_{0}}{\sqrt{{\left(x\;-\;x_{0}\right)}^{2}\;+\;{\left(y\;-\;y_{0}\right)}^{2}\;+\;f^{2}}}\right)$$

**Figure 3:**
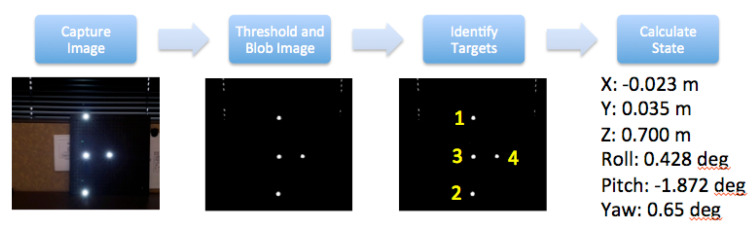
SVGS algorithm flow [7].

**Implementation of the SVGS Algorithm.** The SVGS calculation begins with the capture by the smartphone camera of the illuminated pattern on the target spacecraft. The image is then processed: the image is first converted to a binary image using a specified brightness threshold value. Blob extraction is performed on the binary image to find all bright spot locations. Location, size and shape characteristics of the blobs are captured. Depending on whether there are any other objects in the field of view that may generate bright background-noise spots, the number of blobs may exceed the number of targets. To account for any noise and to properly identify which target is which, a subset of four blobs is selected from among all that are identified, and some basic geometric alignment checks derived from the known orientation of the targets are applied. This process is iterated until the four targets have been identified and properly labeled. The target centroids are then fed into the state determination algorithms. Using the collinearity equation formulation, the relative state is determined using a least-squares procedure. The SVGS algorithm flow is shown in Figure 3 [7].

## 2. Materials and Methods

### 2.1. SVGS Coordinate System and Targets

**Figure 4:**
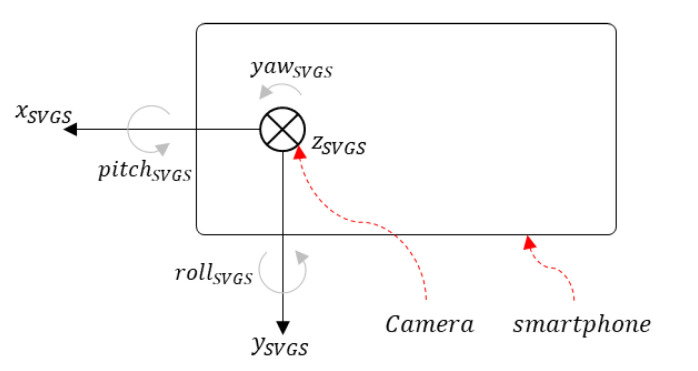
The SVGS coordinate system is defined relative to the camera location on the smart phone.

**Figure 5:**
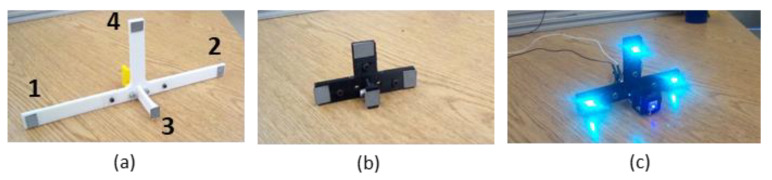
SVGS targets: (**a**) retroreflective target R1, (**b**) smaller retroreflective target R2 and (**c**) LED target.

SVGS calculates the position and attitude of an illuminated target relative to a coordinate system attached to the smartphone’s camera, as shown in Figure 4. The *Z* axis is orthogonal to the camera plane following the right-hand rule, and the roll axis (*θ*) denotes planar rotation around the *Y* axis. The assessment tests are aimed at quantifying the accuracy of the SVGS position and attitude estimates for three types of moving targets in three axes of motion (*X*, *Z* and *θ*). Figure 5 shows the three different SVGS targets used in this study.

For R1 and R2, SVGS uses the camera flash to illuminate the targets, while in the LED target, the flash is deactivated. Different types of SVGS targets can be better suited for different applications, and LED targets enable the future deployment of SVGS in platforms other than a smartphone.

This study investigates the effects of target geometry, target speed and type of target on SVGS performance. Table 1 shows the dimensions of the SVGS targets (Figure 5), as the distance of each reflective point to the center of the bracket, following the target coordinate system (Figure 1, left). 

### 2.2. Overview of SVGS Motion Tests

**Figure 6:**
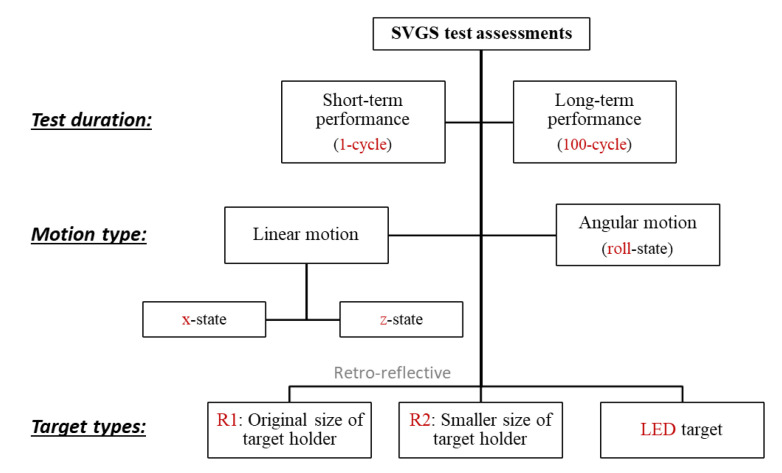
Test matrix for SVGS accuracy assessment tests.

Figure 6 shows an overview of the assessment tests performed. The short-duration (single cycle) tests estimate the semi-static accuracy of the measurements, while the long-duration tests (100 cycles) were intended to estimate the statistics of the measurement error. In the short-duration tests, the SVGS targets were moved following a single cycle of a sinusoidal motion profile provided by a computer-controller motion stage with encoder feedback. The targets were moved following a single axis of motion at a time, while the remaining 5-DOFs were kept constant. Both the *x*- and *z*-axes of the SVGS measurements were tested, as well as roll-state (single-axis rotation).

### 2.3. Assessment of Linear Motion Measurements

**Figure 7:**
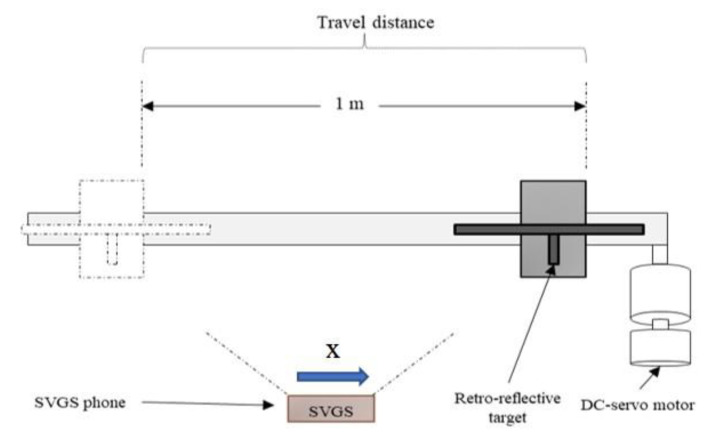
Layout of the *x*-axis SVGS assessment tests.

**Figure 8:**
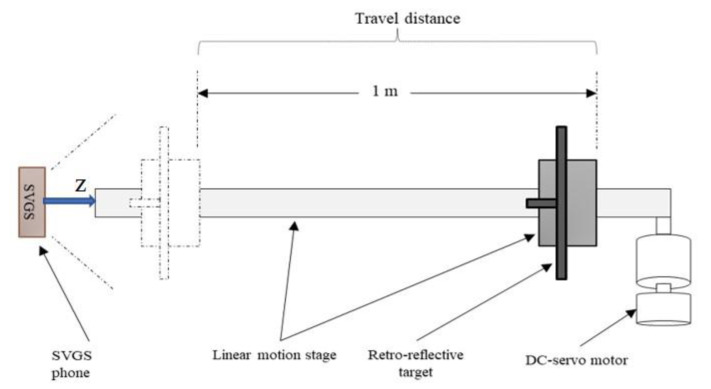
Layout of the *z*-axis SVGS assessment tests.

For all the assessment tests, the SVGS device was fixed to ground, while the targets were placed in a computer-controlled motion stage. A desired motion profile can be generated, and the corresponding encoder measurements compared to the SVGS output. The motion stage was driven by a Parker Hannifin ERV56 linear rodless actuator connected to a 3-phase brushless DC servo motor with a built-in incremental encoder with an accuracy of ±2 arc min, used to determine the position of the target in the motion stage—encoder readings are considered to describe the “true” position of the target. Figure 7 and Figure 8 illustrate the test setups to assess SVGS accuracy in both the *x* and *z* states, respectively. In the *x*-axis tests, the SVGS targets move along a line parallel to the *XY* plane of the smartphone’s coordinate system, while for the *z*-axis tests, the targets move along a line orthogonal to the *XY* plane of the smartphone. Both tests follow a sinusoidal motion profile with a 1 m peak-to-peak amplitude. To assess the effect of target velocity on SVGS performance, several frequencies of the sinusoidal motion profile were used: 0.2, 0.1, 0.05 and 0.008 Hz. The frequencies and range of motion for these tests were suggested by our NASA MSFC collaborators to cover the typical range of target speeds in common spacecraft/CubeSat proximity operations where SVGS could be used.

**Figure 9:**
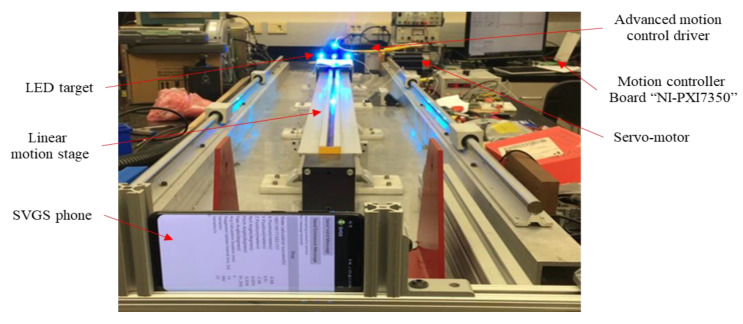
Experimental setup for assessment of linear motion tests (*z*-axis).

Figure 9 shows the experimental setup used to achieve precise motion profiles using a linear motion stage. The motion stage is controlled by a real-time National Instruments motion controller (NI-PXI-7350, Budapest, Hungary), which drives a DC motor with a built-in high-resolution encoder. Encoder measurements are used as reference signal for the actual motion of the carriage, which can then be compared to SVGS data.

### 2.4. Assessment of Angular Motion Measurements

**Figure 10:**
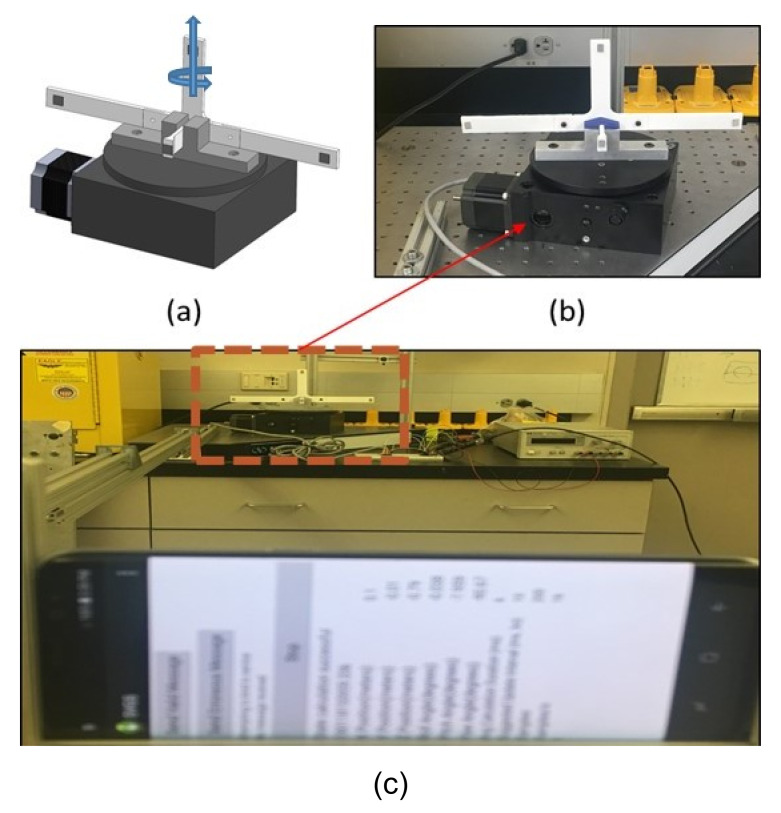
Angular motion testbed: (**a**) roll axis, (**b**) rotational platform and (**c**) experimental setup.

The assessment of SVGS performance in measuring target rotational motion around the roll axis was performed using a computer-controlled rotary motion stage as shown in Figure 10. The rotary motion stage (Figure 10a) includes a 1:180 gear reduction, providing an angular resolution of 0.01°. The SVGS smartphone was placed 2 m away from the motion stage holding the target (Figure 10c).

At the start of a rotation-motion test, the plane of the smartphone camera is parallel to the plane of the reflective target (Figure 10c), which corresponds to a roll angle equal to zero. The x-position of the target (as measured by SVGS) is initially set to zero, i.e., the smartphone camera is coaxial with the out-of-plane leg of the SVGS target. All other SVGS states remain constant during these tests.

## 3. Results

### 3.1. Linear and Rotational Motion Assessment Tests

**Figure 11:**
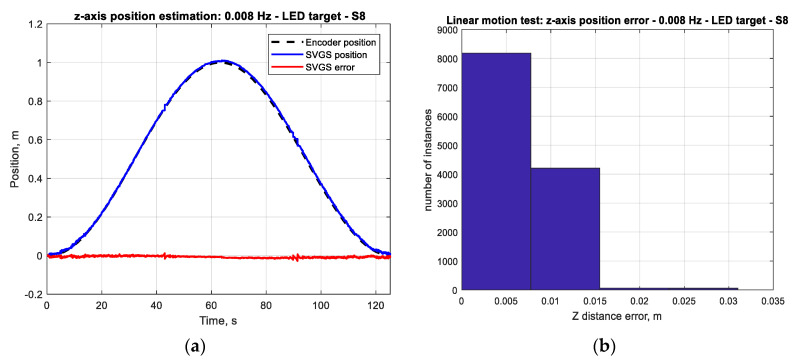
(**a**) Actual motion and SVGS position estimates (*z*-axis); target moving at 1 m p–p sinusoidal at 0.008 Hz. (**b**) Histogram of position error (*z*-axis); target moving at 1 m p–p sinusoidal at 0.008 Hz.

Figure 11a shows the actual motion of the carriage during a single-cycle test (1 m peak-to-peak sinusoidal at 0.008 Hz) as measured by the encoder, along with the corresponding SVGS position estimation for the *z*-axis. Figure 11b shows a histogram of the position error (difference between the reference motion and SVGS measurements), showing that at this target speed, the position errors in the *z*-axis stay within 1% of the range of motion. 

**Figure 12:**
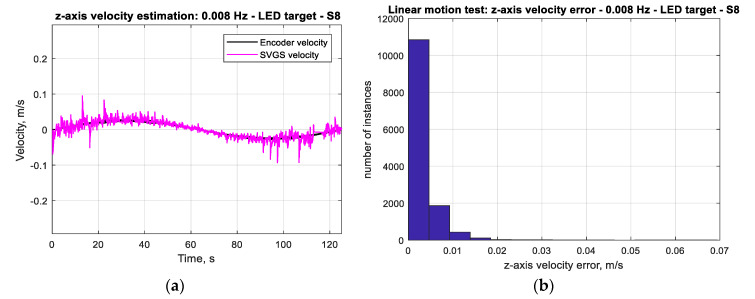
(**a**) Actual velocity (measured by encoder) and SVGS velocity estimates (*z*-axis) for target moving at 1 m p–p sinusoidal at 0.008 Hz. (**b**) Histogram of velocity error (*z*-axis) for target moving at 1 m p–p sinusoidal at 0.008 Hz.

**Figure 13:**
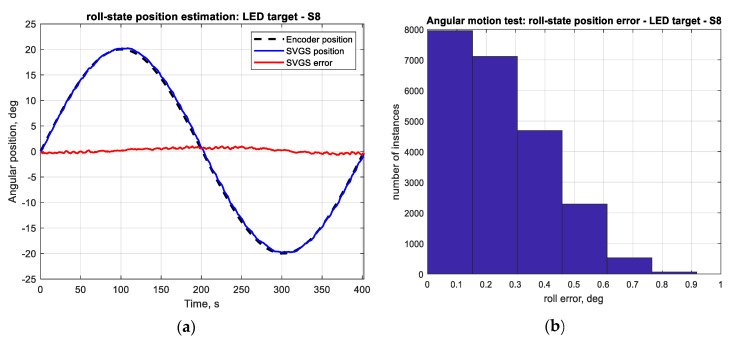
(**a**) Angular position measured by encoder and SVGS-based angular-position estimate (roll-axis). (**b**) Histogram of angular-position measurement error (roll-axis).

**Figure 14:**
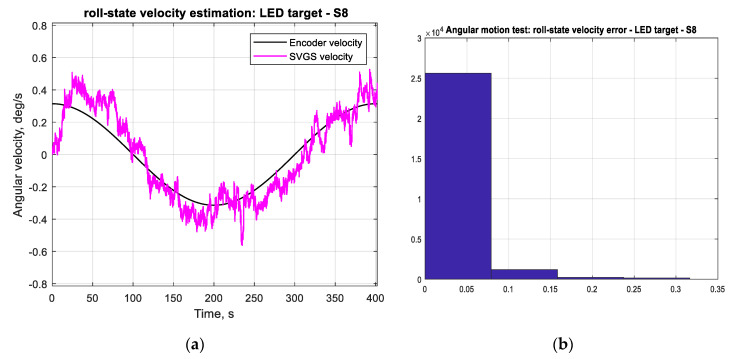
(**a**) Angular velocity measured by encoder and SVGS-based angular velocity estimate in roll-axis. (**b**) Histogram of angular-velocity estimate error in roll-axis.

The accuracy of the SVGS-based linear velocity estimation for the same motion profile was investigated. Figure 12a compares the SVGS-based velocity estimate with the actual velocity of the moving target. The SVGS-based velocity estimate is based on finite differences, and a histogram of the velocity error (Figure 12b) enables estimation of the noise statistics. For the long-duration performance assessment, 100-cycle tests are used since long-duration stability is critical for the successful use of SVGS in satellite or spacecraft applications. The long-duration tests (100 cycles) were used to estimate the statistics of the measurement error by measuring the distribution of the measurement errors as shown in Figure 11b, Figure 12b, Figure 13b and Figure 14b.

Figure 14 shows the SVGS-based angular velocity estimates with the given angular motion profile shown in Figure 13. The angular velocity estimate is based on finite differences followed by a first-order digital low-pass filter. While SVGS is adequate for estimating angular position (attitude), a different sensor such as a MEMS gyro is a better choice for estimating angular rate of rotation.

### 3.2. Effect of Sampling Rate on SVGS Performance

**Figure 15:**
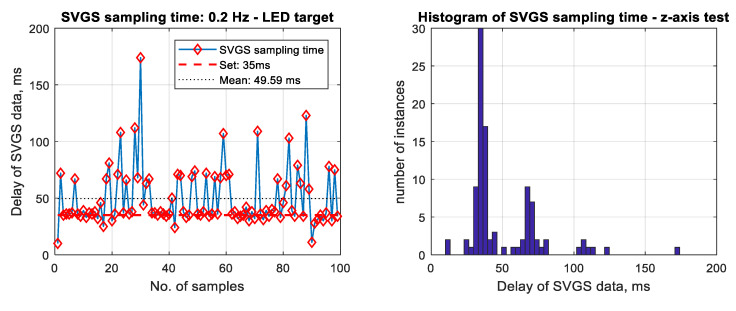
Actual SVGS sampling time (**left**), and histogram plot of sampling time distribution (**right**).

Latency has significant impact in vision-based positioning systems due to the time needed for image acquisition and image processing [7,8]. Several factors affect the sampling time of a vision-based system, such as the available processor power, complexity of the acquired image and type of target. In its current version, SVGS is an Android application, and as such, it does not run in real time due to fluctuations in CPU load that lead to non-deterministic sampling times, as shown in Figure 15, where the plot on the left shows number of samples versus the time between samples, while the figure on the right shows the corresponding histogram of sampling time. This sample test was conducted with the LED target and 0.2 Hz motion profile (*z*-axis). The means of the SVGS sampling times for the three different targets and motion velocities are shown in Section 3.3.

### 3.3. SVGS Error Statistics

**Figure 16:**
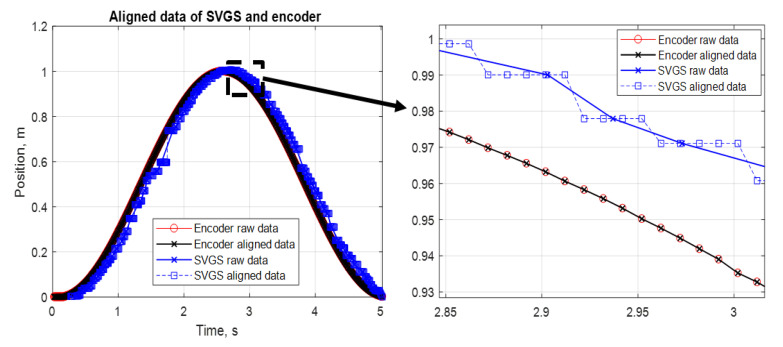
Time-aligned SVGS and encoder data (**left**), as shown in the zoomed area (**right**).

For error assessment purposes, the varying sampling rate of SVGS requires additional processing before direct comparisons between SVGS and encoder data for error analysis can be made. SVGS data were processed off-line to ensure the alignment of the SVGS and encoder samples in the time domain. The Dynamic Time Warping tool in Matlab’s [16] Signal Processing Toolbox was used to provide equally spaced data points for both measurements, as shown in Figure 16.

Encoder data is acquired at a fixed rate (100 Hz), while SVGS data are available with time stamps but do not follow uniform sampling, as shown in Figure 16. Interpolation in time and space is performed on SVGS data to achieve time aligned data at the same sampling rate as the encoder, enabling the meaningful computation of error statistics. Pre-processing reflects the way in which SVGS data are used, not the way in which the data becomes available. Figure 16 (right) illustrates the use of time warping to yield time-aligned data: time alignment enables computation of positioning error by subtracting SVGS measurements from encoder measurements at the same time samples. 

**Figure 17:**
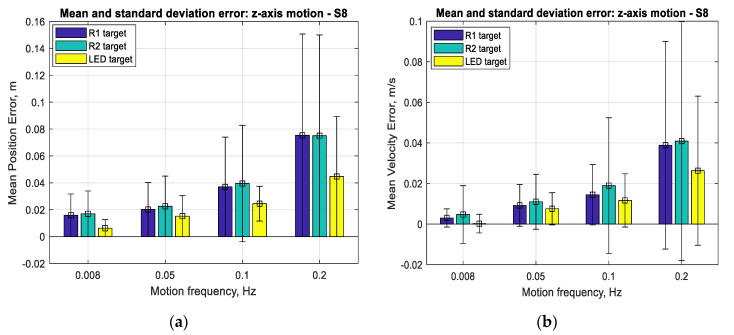
(**a**) Mean error and standard deviation in linear position, *z*-axis. (**b**) Mean error and standard deviation in linear velocity estimation, *z*-axis.

**Figure 18:**
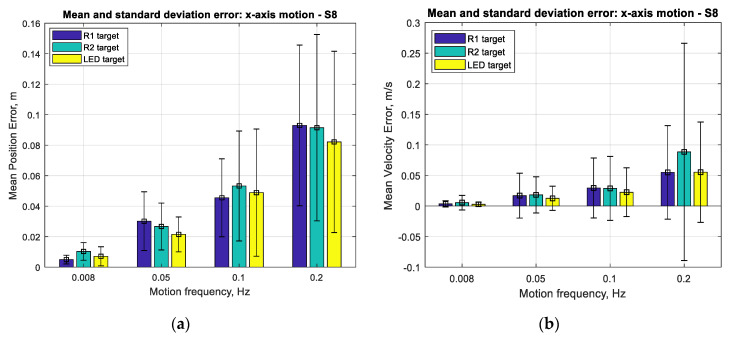
(**a**) Mean error and standard deviation in linear position, *x*-axis. (**b**) Mean error and standard deviation in linear velocity estimation, *x*-axis.

**Figure 19:**
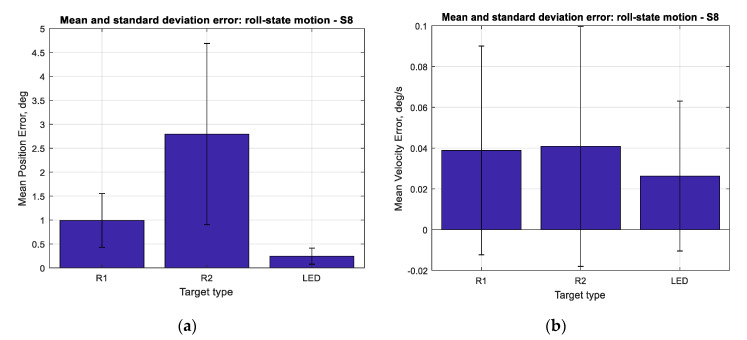
(**a**) Mean error and standard deviation in angular position, roll-axis. (**b**) Mean error and standard deviation in angular velocity estimation, roll-axis.

**Figure 20:**
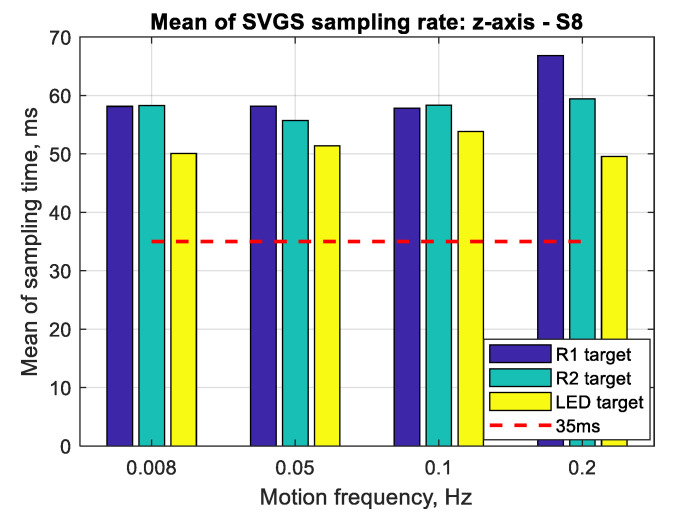
Mean of SVGS sampling rate for various SVGS targets and target speeds.

The results for the performance of SVGS deployed on a Samsung S8 smartphone for three different targets (R1, R2 and LED) at different target velocities (0.2, 0.1, 0.05 and 0.008 Hz at 1 m amplitude) for linear and angular motion tests are shown in Figure 17, Figure 18 and Figure 19. Performance is shown as the mean positioning and velocity error, and the thin bars show the corresponding error distribution (spread) over ±1 standard deviation around the mean error. Figure 20 shows the mean SVGS sampling rate for various SVGS targets and target speeds. 

## 4. Discussion

The assessment of SVGS presented in this paper focuses on its use as a feedback sensor in real-time motion control applications, where time samples are evenly spaced. In such applications, SVGS data are used in equally spaced time samples, even though the SVGS native update rate is uneven when implemented on an Android platform. 

Figure 17 and Figure 18 show that the mean positioning error for all types of SVGS targets increases with target velocity, as expected. LED targets consistently show smaller positioning errors compared to retro-reflective targets. The same can be said from the corresponding errors in linear velocity estimates, as expected. Figure 19 shows the mean errors and standard deviations of the angular position measurements and angular velocity estimates. Angular motion tests also show substantially better performance when using LED targets (a mean error of less than 0.25°) compared to retroreflective targets (a mean error of 1° for the R1 target). 

With respect to target size, the main effect on error is given by the overall size of the target holder: the R2 target has a consistently larger mean error for angular position estimates compared to the R1 target. The means of errors in Figure 17 and Figure 18 seem large when expressed in meters but are more meaningful (and much smaller) when expressed as percentages of the actual measurements (see Figure 11a and Figure 13a, for example). While the errors seem more meaningful when expressed as percentages of the measurements, the error statistics (in meters) are what is needed for use in an SVGS-based Kalman estimator. For this reason, the error results are presented in meters, and not as percentages.

Figure 20 shows the mean SVGS sampling time for different motion velocities and SVGS targets. The test was conducted at 35 msec update rate in the SVGS software: the intention was to identify whether a relationship existed between the target velocity and target type, and the convergence and average sampling rate of SVGS. No significant dependency is found between the sampling time and different motion profiles or types of targets; however, LED targets consistently converge faster and therefore provide a faster sampling rate compared to retro-reflective targets (R1 and R2).

## 5. Conclusions

The purpose of this paper is two-fold. First, it set out to introduce SVGS to the technical community as a relative position and attitude sensor that can be used for a variety of robotic proximity operations (docking, landing and rendezvous) and not just in space guidance and control, as AVGS has been. Second, the paper describes in detail the error statistics of SVGS, as necessary building blocks for estimating noise and covariance matrices for SVGS. These are fundamental tools for the incorporation of SVGS in motion control systems via Kalman filter and LQG control design methods. 

SVGS is a stand-alone relative position and attitude sensor that holds great promise for proximity operations in small satellite applications, such as relative navigation, rendezvous and docking. SVGS provides estimates of the 6DOF position and attitude vector of a target relative to the camera coordinate frame, at sampling rates as high as 35 ms, when deployed on a Samsung S8 smartphone. Since SVGS is a vision-based technique, the error is, of course, dependent on range and camera resolution. To scale the results of this paper to other geometries and camera resolutions, the blob size (the light markers identified in the images) must be scaled with the total resolution (number of pixels) in the image.

This paper provides a quantitative assessment of its performance in terms of accuracy (mean error) and precision (standard deviation). Different factors that affect precision and accuracy were illustrated, such as the effect of the target’s motion profile and the size and type of SVGS targets. The effect of these factors on the sampling time (speed of convergence) of SVGS was also assessed. The exact control of the sampling time is not possible on a sensor that runs on a hardware platform that does not provide strict control of timing (such as most operating systems), but this may not be a critical issue in satellite proximity operations since the target speed is usually small compared to a sensor update rate in the order of 10 Hz or more. The performance of SVGS as a real-time sensor for motion control and the relative navigation of small satellites can be improved based on the specifics of a given application (range, target speed and target size). Future work includes the deployment, testing and assessment of SVGS as motion-control and navigation sensors on other platforms such as single-board PCs and other Android-based systems, thereby eliminating the need for using smartphones for implementing SVGS. SVGS is envisioned to be deployable either on inexpensive, compact platforms (such as the Raspberry Pi 4 or Beaglebone boards) or as a software-based sensor on the user’s platform, such as the guest scientist platform (HLP) on NASA’s Astrobee free-flying robot onboard the ISS, where SVGS can be deployed using both the camera and processor board that are part of the HLP.

The analysis of the error statistics of SVGS presented in this paper enables the incorporation of SVGS as part of a multi-DOF motion-control system by using SVGS error statistics to synthesize a Kalman estimator, which can be part of an advanced control system for navigation and guidance.

## Figures and Tables

**Table 1:** Dimensions of SVGS targets. The target coordinate system is defined in Figure 1, left.

Target Type	Target Number	X (m)	Y (m)	Z (m)
R_1_	1	0	0.143	0
	2	0	−0.143	0
	3	0	0	0.051
	4	0.094	0	0
R_2_	1	0	0.041	0
	2	0	−0.041	0
	3	0	0	0.022
	4	0.036	0	0
LED	1	0	0.055	0
	2	0	−0.055	0
	3	0	0	0.037
	4	0.048	0	0
